# Near infrared hyperspectral dataset of healthy and infected apple tree leaves images for the early detection of apple scab disease

**DOI:** 10.1016/j.dib.2017.12.043

**Published:** 2017-12-21

**Authors:** Maroua Nouri, Nathalie Gorretta, Pierre Vaysse, Michel Giraud, Christian Germain, Barna Keresztes, Jean-Michel Roger

**Affiliations:** a CTIFL, Interprofessional Technical Center for Fruit and Vegetables, 24130 Prigonrieux, France; b ITAP, Irstea, Montpellier SupAgro, Univ Montpellier, Montpellier, France; c University of Bordeaux, IMS UMR 5218, 33400 Talence, France

## Abstract

This dataset presents two series of hyperspectral images of healthy and infected apple tree leaves acquired daily, from two days after inoculation until an advanced stage of infection (11 days after inoculation). The hyperspectral images were calibrated by reflection correction and registered to match the geometry of one reference image. On the last experiment day, scab positions are provided.

**Specifications Table**

**Table:** 

Subject area	*Biology; Hyperspectral imaging*
More specific subject area	*Plant sciences; Disease detection*
Type of data	*Tables, images (ENVI: Environment for Visualizing Images format)*
How data was acquired	*Images were acquired using a NIR push-broom Hyspex SWIR (Shortwave Infrared)-320-e camera NEO (Norsk Elektro Optikk, NORWAY).*
Data format	*Raw, Analyzed*
Experimental factors	*Images were calibrated by reflection correction and registered to match the geometry of one reference image.*
Experimental features	*Infected and healthy images of apple tree leaves were acquired daily using NIR hyperspectral camera. Inoculated plants were assessed daily by visually rating the development of scab symptoms by an expert.*
Data source location	*Bergerac, France*
Data accessibility	*The data is available with this article*

**Value of the Data**

• The data provide a spatial, spectral, and temporal tracking of apple scab disease in a hyperspectral image sequence. Thus, they allow any other researcher to perform chemometric analyses for early disease detection.
• The images provided were registered so that pixel positions match between dates.

• The data could be used to test image processing methods for pathogen detection at early or later stages of the infection.
• The data were acquired using NIR hyperspectral camera and they could be compared to other disease detection measurements [1].
• The data were acquired at leaf-level. Thus, they can be used to establish a link with plant physiology studies.

## 1 Data

Data provided in this article relate to two series of hyperspectral images of healthy and inoculated apple tree leaves acquired daily, from day 2 to day 11 after inoculation using NIR hyperspectral camera. Due to weekend lag, images are lacking on days 7 and 8 after inoculation. Apple scabs become visible to the naked eye at day 9 after inoculation.

**Fig. 1:**
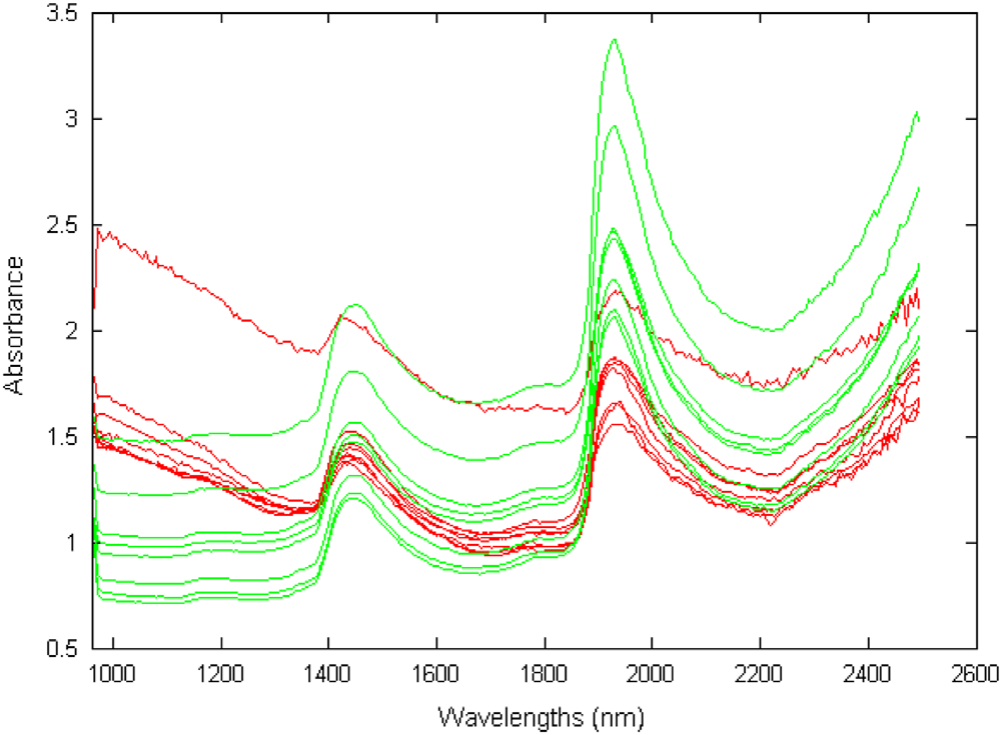
Absorbance spectral curve of healthy (green) and infected (red) extracted spectra.

**Fig. 2:**
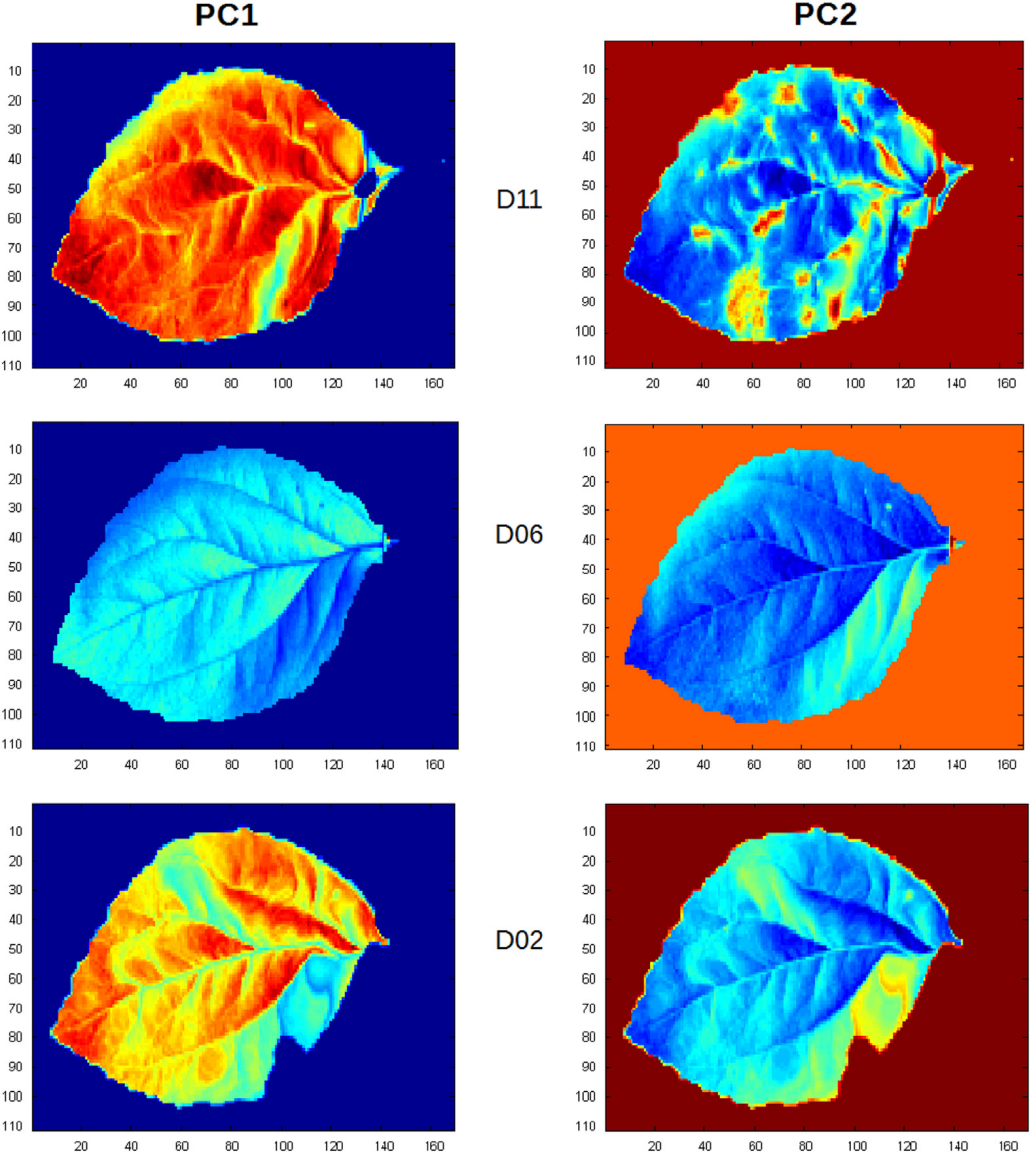
Scores of the principal components 1 and 2 of the PCA tested on days 11, 6 and 2 of the infected leaf images.

**Fig. 3:**
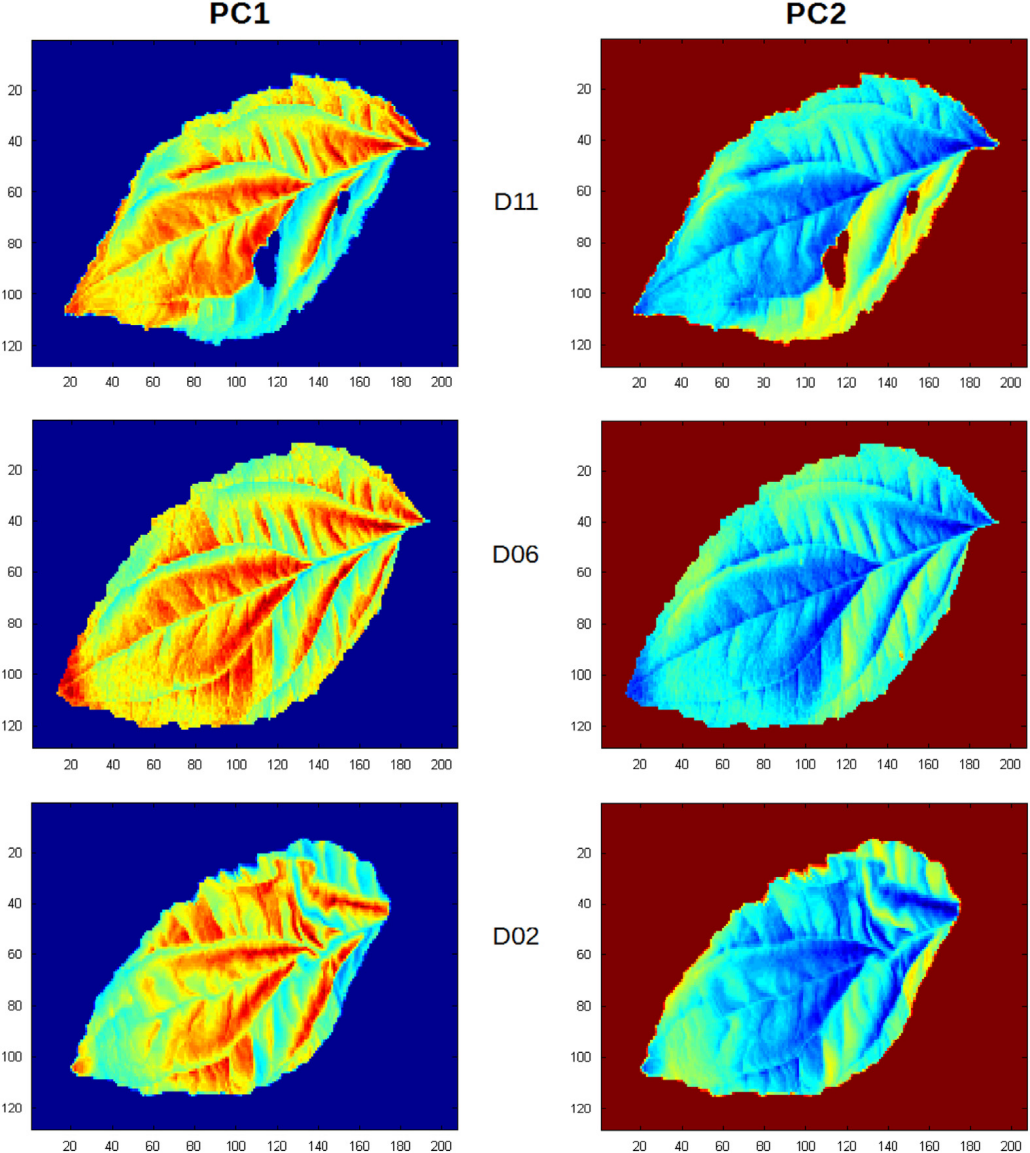
Scores of the principal components 1 and 2 of the PCA tested on days 11, 6 and 2 of the healthy leaf images.

Eight spots were extracted from both the infected and healthy leaves at day 11 after inoculation. Mean spectrum was calculated for each spot, providing 8 spectra from infected spots and 8 spectra healthy ones. Spectra were preprocessed by applying the logarithm, yielding those presented Fig. 1. Principal Component Analysis PCA was calculated on these spectra using Matlab software R2012b and tested on hyperspectral images at three stages of the infection process (days 11, 06, 02) (Fig. 2, Fig. 3).

## 2 Experimental design, materials, and methods

### 2.1 Treatments

Two apple seedlings were grown in pots and used on the experimentation. The apple cultivar selected was Golden Delicious Reinders®. One of the plant seedlings was inoculated through spray-inoculation with a suspension of 5·10^5^ conidiospores per milliliter [2]. The other plant was kept healthy and sprayed with water. Plants were incubated in an experimental greenhouse to establish a controlled environment for the development of the fungus. They were irrigated and fertilized as required [3]. On the last experiment day, inoculated leaves were examined by an expert under the magnifying glass in order to identify scab spot positions.

### 2.2 Data collection

**Fig. 4:**
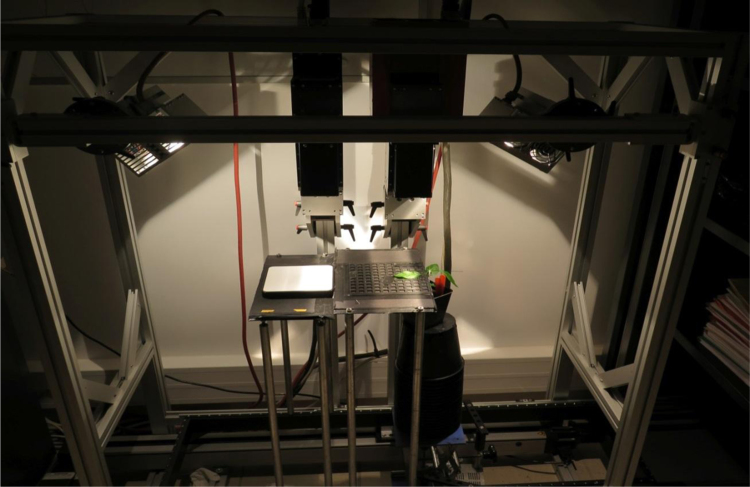
Hyperspectral image acquisition device.

Close range hyperspectral images of healthy and infected leaves were acquired daily under laboratory conditions using HySpex SWIR-320m-e camera (Fig. 4). The camera acquired successive lines with a step of 0.287 mm. Each line was made up of 320 pixels and was 0.287 mm in width. Each pixel was made up of 256 spectral bands ranging from 960 to 2490 nm with a 6 nm spectral sampling interval. The lighting was provided by a halogen source. The incoming halogen irradiance was estimated on a line-by-line basis by using a 99% diffuse reflectance panel (Spectralon®, Labsphere) horizontally placed next to the acquired scene and used for the image reflectance correction. The leaf held over a magnetized plate was fixed using a thin iron bars.

### 2.3 Analysis

Images were calibrated to spectral radiance by the reflectance correction. A mask was applied to remove the image background and the leaf edge. Then, images were registered to match the image acquired at the day six after inoculation [4]. This Registration was performed using a registration estimator application implemented a commercial software (Matlab R2017a, the MathWorks Inc., Natick, MA).
